# Supplementary material for: FastqPuri: high-performance preprocessing of RNA-seq data
Source: BMC Bioinformatics. 2019 May 3;20:226. doi: 10.1186/s12859-019-2799-0 (PMC6500068; doi:10.1186/s12859-019-2799-0)
Supplement: Supplementary file 2 — Archive of FastqPuri. Archive containing all files needed to install and run FastqPuri v1.0.6. Date stamp March 22, 2019. (GZ 47,819 kb) [file 12859_2019_2799_MOESM2_ESM.gz › FastqPuri-1.0.6/html/struct__fa__data-members.html]

FastqPuri: Member List


FastqPuri

\_fa\_data Member List

This is the complete list of members for \_fa\_data, including all inherited members.

|  |  |  |
| --- | --- | --- |
| entry | \_fa\_data |  |
| entrylen | \_fa\_data |  |
| linelen | \_fa\_data |  |
| nentries | \_fa\_data |  |
| nlines | \_fa\_data |  |


---

Generated on Mon Mar 19 2018 23:42:01 for FastqPuri by  

 1.8.14
